# A Community-Informed Approach to COVID-19 Vaccine Roll-Out in Under-served Areas in Chicago

**DOI:** 10.3389/fpubh.2022.863125

**Published:** 2022-06-20

**Authors:** Laura DiVirgilio, Arianna Boshara, Bijou R. Hunt, Jacquelyn Jacobs, Kate Just, Amy K. Johnson

**Affiliations:** ^1^Chicago Medical School, Rosalind Franklin University of Medicine and Science, North Chicago, IL, United States; ^2^Sinai Infectious Disease Center, Sinai Health System, Chicago, IL, United States; ^3^Sinai Urban Health Institute, Sinai Health System, Chicago, IL, United States; ^4^Department of Pediatrics, Northwestern University, Feinberg School of Medicine, Chicago, IL, United States; ^5^Department of Pediatrics, Ann & Robert H. Lurie Children's Hospital of Chicago, Chicago, IL, United States

**Keywords:** COVID-19, vaccine, vaccine distribution, equity, community-informed

## Abstract

The availability of the COVID-19 vaccine in the US created an urgent need for strategies to achieve widespread vaccine distribution, but approaches to achieving equitable distribution, including reaching communities of color, varied across the country. To add to the knowledge base around targeted vaccine roll-out among underserved communities, the current study presents results from patient vaccination data and staff interviews conducted at Sinai Chicago, a safety-net healthcare system serving under-resourced communities. A total of 11,313 patients received at least one dose of Pfizer or Moderna COVID-19 vaccine between January and October 2021 at a Sinai Chicago facility. The sample was primarily comprised of Hispanic and non-Hispanic Black persons, with a mean age of 47 years, and was split evenly between female and male individuals. Compared to non-Hispanic White persons, Hispanic persons were 1.4 times more likely to have completed the full course of vaccination, while non-Hispanic Black persons were 40% less likely. People ages 18–24 were less likely to be fully vaccinated compared to all other adult age groups. Compared to privately insured persons, publicly insured persons were 40% less likely to have been fully vaccinated. The vaccine roll-out approach focused on educating the community through town halls and targeted messaging to address common myths and misconceptions about the vaccine, as well as developing the necessary infrastructure to administer the vaccine in a variety of community settings. This study illustrates COVID-19 vaccine roll-out in an under-resourced urban area in Chicago and provides insight on future implementation of vaccine intervention in hard to reach communities.

## Introduction

The availability of COVID-19 vaccines in December 2020 prompted national vaccination strategies across the United States (U.S.) to ensure adequate access and optimal coverage. As of January 11, 2022, 66.6% of the total eligible U.S. population was fully vaccinated (1). Several studies reveal differences in vaccination rates among age groups (2), racial/ethnic identity (3, 4), and socioeconomic status (5) among other traits. Vaccination rates are highest among non-Hispanic whites and lowest among Black and Latinx populations. Vaccine hesitancy is highest among Black and Latinx communities for a multitude of reasons including medical mistrust due to systemic racism (6, 7). There is evidence that integrated community-based interventions are effective in increasing childhood vaccine series completion, and adult influenza and pneumococcal vaccination rates in target populations, including communities with high rates of vaccine hesitancy and those that are predominantly Black and Latinx (8). However, we are unaware of any studies documenting the implementation and evaluation of community-based approaches to overcome hesitancy and achieve equitable vaccination during the COVID-19 vaccine roll-out.

During the COVID-19 vaccine roll-out the United States Centers for Disease Control and Prevention (CDC) advised that vaccination prioritization be stratified first by age (>65 years old) and occupational risk based on recommendations by the Advisory Committee on Immunization Practices (ACIP) (9). These recommendations were met with criticism, including that the “race blind approach” fueled inequities in vaccine uptake (10). Ultimately, implementation of the vaccine roll-out was determined at the state level, which led to some variation across the country. In Chicago, the COVID-19 vaccine roll-out has been conducted in four eligibility phases beginning with Phase 1a on December 15, 2020. Eligible persons in Phase 1a included residents and staff of long-term care and other residential facilities, as well as health care workers. Phases 1b and 1c expanded eligibility to elderly individuals (>65 years old) and those with underlying conditions, respectively. In February 2021, Chicago launched Protect Chicago Plus, an initiative to remove barriers and increase vaccination rates among residents in community areas that experienced the highest burden of disease. In 13 community areas within the city, all restrictions on eligibility were lifted aside from residency. Finally on April 19, 2021, Phase 2 expanded eligibility to all individuals ages 16 and older (11).

Beyond the ACIP recommendations to prioritize vaccination by age and occupation, the systemic lack of infrastructure in historically disinvested communities also contributed to lower COVID-19 vaccination rates due to decreased access in communities of color (12). In order to overcome lack of access, alternative methods including in-home vaccination, establishing mass vaccination locations, canvassing door-to-door, and increasing availability of community pharmacy based vaccination have been utilized (13, 14).

Sinai Chicago (Sinai) is an urban safety-net hospital serving predominantly Black and Latinx communities on the West and Southwest sides of Chicago. Leveraging longstanding relationships and expertise working with communities to improve health outcomes, Sinai developed a vaccine distribution approach to mitigate hesitancy and increase vaccine rates among communities of color. This study uses a mixed-methods approach to describe the health system's locally tailored approach to COVID-19 vaccine distribution to overcome vaccine hesitancy and resistance among West and Southside Chicagoans. Quantitative methods were used to characterize the population vaccinated at Sinai and qualitative methods were used to examine the roll out of one COVID-19 vaccination program, including successes and challenges of Sinai's efforts.

## Materials and Methods

### Setting

Sinai Chicago is Illinois' largest private safety-net health system, serving the West and Southwest sides of Chicago. These under-resourced communities are made up of predominantly racial and ethnic minority populations, which suffer a disproportionate burden of chronic disease morbidity and mortality (15).

Sinai Chicago includes Mount Sinai Hospital (MSH), Holy Cross Hospital (HCH), and Schwab Rehabilitation (SRH). MSH is a 319-bed acute care community teaching hospital and an adult Level 1 trauma center; HCH is a 264-bed community hospital; SRH is a 102-bed rehabilitation hospital. MSH and HCH have Emergency Departments (ED) and all three facilities offer a range of inpatient and outpatient services. Through Sinai Medical Group (SMG), Sinai Chicago also operates 14 outpatient clinics across the system's service area and includes a range of services including COVID-19 testing.

The majority of persons in the Sinai Chicago service area, as well as patients seen at Sinai Chicago, are un- or under-insured, non-Hispanic Black or Hispanic, and many are Spanish-speaking only (15). The Mount Sinai Hospital Institutional Review Board approved this project (protocol #21–47).

### Study Design

To describe and enhance our understanding of the hospital's approach to COVID-19 vaccine distribution, we conducted a mixed-methods study. This study combined results from a cross-sectional quantitative analysis of hospital vaccination data with semi-structured interviews of key hospital staff and community benefits staff. Our intent was to use these two data sources to complement and enhance our understanding of the hospital's vaccine roll-out approach. The Mount Sinai Hospital IRB approved this study (#21–47).

### Sample Selection

Qualitative data were obtained *via* semi-structured key-informant interviews (interviews). A purposive sample was constructed that consisted of leaders of divisions involved in the COVID-19 vaccine roll-out across the health system. Interviews were conducted with a total of 10 individuals from the following divisions and personnel: Marketing—VP Communications & Marketing, System Director of Marketing, Digital Marketing Specialist; Sinai Community Institute (SCI)—Executive Director SCI, Director of Volunteering and Community Services, System Director Multicultural Community Relations; Patient Access/Scheduling—Patient Access Director; Information Systems (IS)—IS Digital Health Manager; Pharmacy—Director of Pharmacy; Infectious Diseases—various physicians.

### Data Collection

#### Quantitative Data

Quantitative data were obtained *via* Sinai Chicago's electronic medical records (EMR). The study population included all Sinai Chicago patients 18 years of age and older who had at least one COVID-19 vaccination (Pfizer or Moderna) between January 1, 2021 and October 31, 2021 in any Sinai Chicago outpatient location.

#### Interview Guide

Through open-ended questions we sought to understand each division's responsibilities in the vaccine roll-out process. Key questions included: describe your department's role in the vaccine roll-out; what were the largest obstacles your department faced; and, is there anything you would change about the approach in the future. Interviews were conducted in English over a HIPAA-compliant teleconference line between August 2021 and September 2021 and each lasted between 30 and 60 min. Interviews were not recorded but notes and thematic summaries were documented. In accordance with the Mount Sinai Hospital IRB, written informed consent was waived.

### Demographic and Outcome Variables

Demographic variables for the vaccinated patient population included sex, age, race/ethnicity, and patient insurance coverage type. Sex was a dichotomous variable coded 1 for patients for whom the EMR indicated male sex at birth and 0 for patients indicated female sex at birth. Age was a categorical variable coded to groups: 18–24 (referent group), 25–34, 35–44, 45–54, 55–64, and 65+. Race/ethnicity was a categorical variable coded to groups based on documentation in the EMR: non-Hispanic White (referent group), non-Hispanic Black, Hispanic, non-Hispanic Asian, and Other/Unknown. Our independent variables were sex, age, race/ethnicity, and insurance coverage. Our dependent variable was vaccination status. For patients missing ethnicity data in the EMR, we undertook the following process to recode data.

First, of the total records (*N* = 11,313) there were 4,137 with Hispanic ethnicity identified in the EMR. For the remaining records (*n* = 7,176), we first applied a surname recode process, which entailed checking the patient's last name against the 1990 US Census list of the 639 most frequently occurring heavily Hispanic surnames (16) and recoding patients with these last names to Hispanic ethnicity (*n* = 707). Second, for those not recoded to Hispanic ethnicity *via* surname, we used the EMR indicator for “patient primary household language is Spanish” to recode to Hispanic ethnicity (*n* = 42). Finally, for anyone not yet recoded to Hispanic ethnicity, we manually searched for and evaluated last names with the letters “ez,” “ll,” or “rr” occurring (16) (section 7.1.3) and recoded these to Hispanic ethnicity (*n* = 28) as appropriate. This brought our total number of records with Hispanic ethnicity to 4,914.

Insurance coverage type was split into four groups: private, public, uninsured, and unknown. Private coverage included commercial, motor vehicle accident (MVA) personal injury, medical assistance no grant (MANG), preferred provider organization (PPO), prompt pay, and work comp. Public coverage included grants, Medicaid, and Medicare. Uninsured coverage included patient pay and charity. Regarding the unknown insurance coverage type, the United States was providing the COVID-19 vaccine for free and therefore many facilities were not asking patients for their insurance information.

Three vaccine outcomes were considered. First dose only captured patients receiving only one injection. Fully vaccinated captured patients receiving both injections. Fully vaccinated by demographic subgroup was a percentage variable that was calculated by dividing the number of total patients who received both doses of the COVID-19 mRNA vaccine by the total number of patients in each group. Demographic subgroups included sex, race, age, and insurance coverage.

Vaccine administration dates were collected between January 1, 2021 and October 31, 2021. Observations were dropped if the patient received their first dose in October 2021 and did not receive a second dose to ensure patients who had their second shot scheduled for after October 1, 2021 were not captured in the “first dose only” group.

### Analysis

A rapid qualitative analysis approach was used to analyze interview data. Interviews were not recorded or transcribed, but at least two project staff recorded detailed notes during each key informant interview. Notes were then collated by one staff member who identified common themes around which to organize the data. Over the course of multiple team meetings, four program staff discussed themes and used an iterative process to finalize the interpretation of findings.

For the quantitative EMR data, we determined whether or not vaccination status (first dose only vs. fully vaccinated) differed by sex, race/ethnicity, age, and insurance coverage by performing chi-square tests for each demographic characteristic to determine whether differences were statistically significant between groups. Next, we ran a binary logistic regression model to determine the association between patient demographic characteristics and fully vaccinated status. The reference group for sex was male; for race/ethnicity was white, for age was 18–24, and for insurance coverage was private insurance coverage. Frequencies were added in Table 1 to describe the proportion of each variable at the univariate level. Quantitative data was analyzed using Stata Version 14.1.

**Table 1:** COVID-19 vaccine patients: demographics by vaccine course status.

	**Total**		**First dose only**		**Fully vaccinated**			***P*-Value**
	***n***	***%***	***n***	***%***	***n***	***%***	***%***	
Total	11,313	100.0	625	5.5	10,688	94.5	94.5	
**Sex**								
Male	5,545	49.0	306	49.0	5,239	49.0	94.5	0.978
Female	5,768	51.0	319	51.0	5,449	51.0	94.5	
**Race/Ethnicity**								
Non-hispanic white	1,470	13.0	78	12.5	1,392	13.0	94.7	0.000
Non-hispanic black	2,511	22.2	213	34.1	2,298	21.5	91.5	
Hispanic	4,930	43.6	196	31.4	4,734	44.3	96.0	
Non-hispanic Asian	842	7.4	41	6.6	801	7.5	95.1	
Other/unknown	1,560	13.8	97	15.5	1,463	13.7	93.8	
**Age**								
Age 18–24	1,118	9.9	89	14.2	1,029	9.6	92.0	0.000
Age 25–34	2,071	18.3	158	25.3	1,913	17.9	92.4	
Age 35–44	2,060	18.2	120	19.2	1,940	18.2	94.2	
Age 45–54	2,072	18.3	102	16.3	1,970	18.4	95.1	
Age 55–64	2,036	18.0	93	14.9	1,943	18.2	95.4	
Age 65+	1,956	17.3	63	10.1	1,893	17.7	96.8	
**Insurance**								
Private	2,940	26.0	82	13.1	2,858	26.7	97.2	0.000
Public	5,788	51.2	368	58.9	5,420	50.7	93.6	
Uninsured	1,380	12.2	51	8.2	1,329	12.4	96.3	
Unknown	1,205	10.7	124	19.8	1,081	10.1	89.7	

## Results

### Qualitative Outcomes

**Figure 1:**
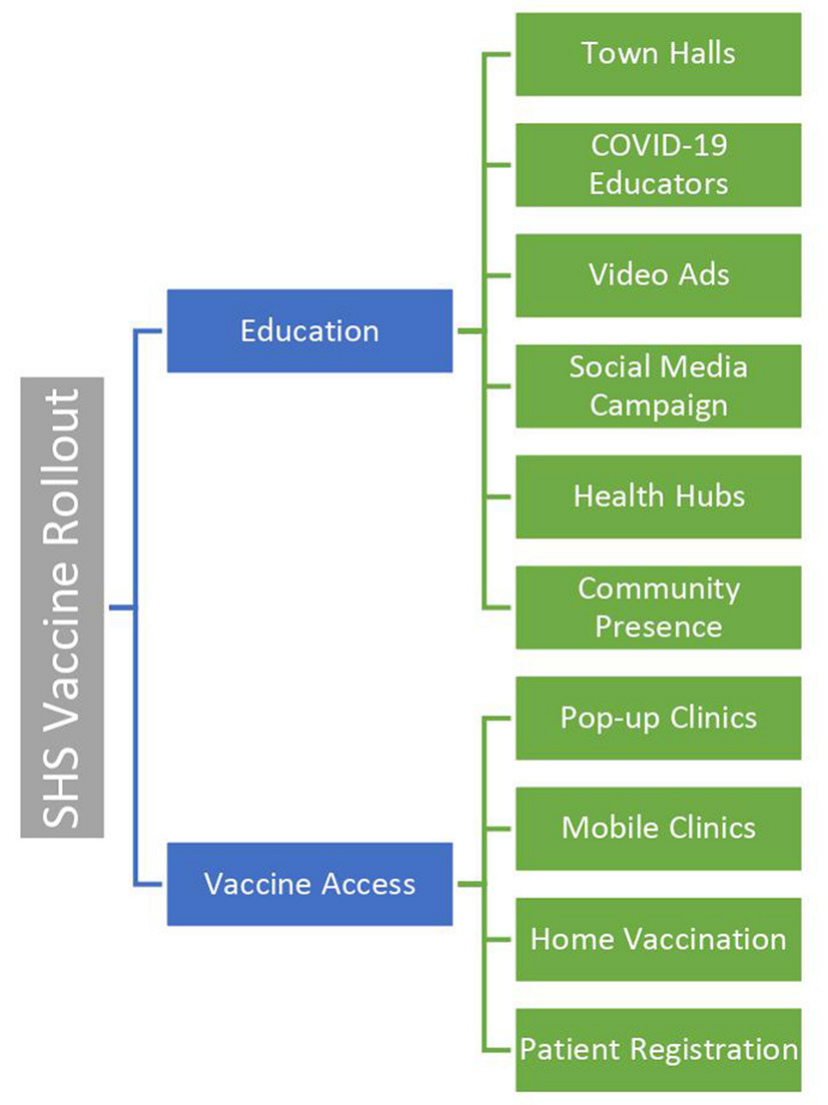
Sinai Chicago vaccine roll-out model.

Sinai Chicago's vaccine roll-out was conducted in two phases: (1) Education; and (2) Access (Figure 1). The first phase, which began in December 2020, focused on explaining the benefits and importance of the vaccine, while simultaneously dispelling myths related to vaccination among community residents. The second phase, which began in January 2021, focused on expanding access to the vaccine. Increased access to vaccination was established by generating interest in appointments, setting up clinics, and establishing the necessary infrastructure to administer vaccines.

### Phase I: Education

#### Marketing

As a first step in developing educational content appropriate for the target communities, the Marketing division worked closely with Sinai Community Institute (SCI), a Sinai affiliate which provides services addressing the social and economic factors impacting the health of our community's most vulnerable members, and a group of Community Health Workers (CHWs). Together, these individuals identified pervasive beliefs and myths in the community (e.g., vaccine causes sterilization; vaccine is a chip implanted to track movement) that would need to be addressed *via* the educational campaign. Additionally, a multicultural marketing company was hired to assist in directing outreach and designing the campaign.

An educational campaign was developed which included a four-part informational video series featuring Sinai's own Infectious Disease physicians and community voices. The series was featured *via* streaming service non-skippable ads, a cost-effective alternative to cable advertisements. The team specified zip code and demographic characteristics for the audience based on Sinai's primary service areas. To further increase distribution, the team created a social media advertising campaign, digital marketing, and advertisements with Univision (an American Spanish-language television network), as well as print ads in local newspapers.

In addition to produced content, the Marketing team launched a social media campaign with the tagline “Why did you get the COVID-19 vaccine?” or “Why are you getting the COVID-19 vaccine?” Driven by this storytelling approach, community members responded with their personal reasons for getting vaccinated, and this was shared broadly on social media. The social media campaign also included digital display ads with content that varied from solely educational (i.e., a link to Sinai's website), to more actionable (i.e., prompting the viewer to complete an online vaccine request form). Additional print materials including flyers and posters, with informational content as well as QR codes linked to videos, were circulated by a street team throughout the community to organizations such as local businesses and churches.

#### Community Benefits and Outreach

Throughout the pandemic Sinai hosted at least one virtual town hall each month. The town halls were highly specific to the needs and concerns of the communities that Sinai serves and featured subject matter experts that were trusted by the community (i.e., clinicians from the community and faith leaders). Providers used language that was understandable for non-healthcare professionals and were prepared to address questions, concerns, and myths in a non-judgmental way.

Previously established community partners and relationships with community leaders enabled SCI to identify where to direct educational efforts to ensure the town halls would be most persuasive and reach the largest audience. For example, knowing the influence of the faith-based community, the team specifically planned the first town hall for local ministers, providing information about the vaccine, myths, and how to access the vaccine through Sinai. Additional town halls targeted groups with high COVID-19 burden or low vaccine uptake, such as seniors and those under 35. These events contributed to establishing Sinai as a COVID-19 resource on the West side of Chicago. In addition to direct interactions, SCI utilized email, text and social media to contact the community.

Sinai Community Institute made substantial efforts to target the youth and young adult population (30 and under). Age-based outreach extended beyond the town halls. SCI leveraged their summer program, One Summer Chicago, to engage the 65 participating teens in researching their own misconceptions about the vaccine. The project gave the teens the opportunity to discuss myths and their own feelings about the vaccine and the pandemic, as well as a chance to present their findings. They then took that that information back to their various communities. SCI also partnered with local schools to host town hall events aimed at youth and their parents to encourage parents to vaccinate their children.

### Phase II: Vaccine Access

#### Appointment Scheduling

One of the first steps taken to promote access to vaccine appointments was the development of an online vaccine request form which captured data on patients interested in receiving the vaccine. Individuals in Sinai's main service areas were prioritized for vaccine appointments by filtering requests by zip code. Staff from the scheduling department followed up to schedule the vaccine appointment. Additionally, SCI also provided facilitated sign up for individuals who required extra support, such as seniors and those with barriers like technology or transportation.

In an effort to increase awareness of this online request form, IS designed a one-time text messaging campaign for existing Sinai patients, providing direct access to the form. The campaign was directed to ambulatory patients with a visit in the last year and who had previously consented to receiving text messages from Sinai's appointment reminder system. About 15,000 patients received a text message in May 2021 reading, “Sinai Chicago is now offering COVID-19 vaccines to all Chicago residents 16 and older, click on the link to submit your COVID-19 vaccine request form.” Embedded in the message was a link that navigated users directly to the vaccination request form on Sinai's website.

Sinai also undertook a more focused approach to scheduling vaccines through outreach to existing outpatients of Sinai Medical Group (SMG). SMG physicians, nurses, and case workers reached out directly to existing eligible patients to provide education about the vaccine and its benefits, and to address any myths or questions that were raised. Patients interested in scheduling a vaccine appointment were referred to Patient Scheduling for follow up and appointment.

#### Pop-Up and Mobile Clinics

Sinai's unique position within communities on the West and Southwest sides of Chicago led to a partnership with the city through Protect Chicago Plus (PCP). As a PCP partner, Sinai hosted pop-up vaccination clinics, the first of which resulted in 500 individuals receiving their first vaccine dose in just 1 day.

Sinai was responsible for all aspects of the operation, including set up, staffing, patient registration, recruitment and administration of the vaccines. Recruitment was achieved with the help of SCI's partners and other local organizations. SCI notified each organization of the event, their time slot, and their capacity. Partnering with these organizations was vital for generating interest among residents as well as guaranteeing follow up when the Sinai team would return to administer second doses. Marketing continued to work alongside the other divisions to increase awareness of clinics through Sinai's website, social media and local print ads. In addition to the pop-up clinics, the city of Chicago provided CTA buses that Sinai converted into mobile vaccination clinics in a similar manner to the pop-up clinics.

### Implementation Challenges

Staffing and funding limitations delayed implementation of aspects of the roll-out, limiting effectiveness. There was a struggle to support the offsite vaccination efforts, and by the time the pop-up clinics were actualized, the demand for them had dwindled as the vaccine was easily accessible. Similarly, having events in the community earlier on in the pandemic would have been more successful. There were many factors involved in these limitations including issues securing vaccines and funding, as well as the pandemic limiting the ability of Sinai staff to be physically in the community interacting with residents.

### Quantitative Outcomes

#### Sample Characteristics

A total of 11,313 patients received at least one shot of the COVID-19 vaccine at Sinai Chicago between January 1, 2020 and October 31, 2021 (Table 1). Of those receiving at least one shot, 51.0% (*n* = 5,768) were female; 43.6% (*n* = 4,930) were Hispanic; 22.2% (*n* = 2,511) were non-Hispanic Black; 13.0% (*n* = 1,470) were non-Hispanic White; 51.2% (*n* = 5,788) were publicly insured, and the mean age was 47.16 years. Of the 11,313 patients vaccinated, 94.5% (*n* = 10,688) had a full dose of vaccination (both the first and second shot).

#### Fully Vaccinated

At the bivariate level, race/ethnicity (*P* = 0.000), age (*P* = 0.000), and insurance coverage were associated with full vaccination (*P* = 0.000) (Table 1). Compared to non-Hispanic White patients, Hispanic persons were 1.4 (*P* = 0.028) times more likely to have completed the full course of vaccination, while non-Hispanic Black persons were 40% (*P* = 0.000) less likely to be fully vaccinated (Table 2). Compared to persons ages 18–24, those aged 35–44, 45–54, 55–64, and 65+ were 1.4 (*P* = 0.021, 1.7 (*P* = 0.001), 1.8 (*P* = 0.000), and 2.6 (*P* = 0.000) times more likely to have been fully vaccinated, respectively. Compared to privately insured patients, publicly insured patients were 40% less likely to have been fully vaccinated (*P* = 0.000).

**Table 2:** Regression analysis.

	**OR (95% CI)**	***p*-Value**
**Sex**		
Male	Ref	Ref
Female	1.0 (0.8–1.2)	0.978
**Race/Ethnicity**		
Non-hispanic white	Ref	Ref
Non-hispanic black	0.6 (0.5–0.8)	0.000
**Hispanic**	1.4 (1.0–1.8)	0.028
Non-hispanic Asian	1.1 (0.7–1.6)	0.648
Other/unknown	0.8 (0.6–1.1)	0.283
**Age**		
Age 18–24	Ref	Ref
Age 25–34	1.0 (0.8–1.4)	0.738
Age 35–44	1.4 (1.1–1.9)	0.021
Age 45–54	1.7 (1.2–2.2)	0.001
Age 55–64	1.8 (1.4–2.4)	0.000
Age 65+	2.6 (1.9–3.6)	0.000
**Insurance**		
Private	Ref	Ref
Public	0.4 (0.3–0.5)	0.000
Uninsured	0.7 (0.5–1.1)	0.109
Unknown	0.3 (0.2–0.3)	0.000

## Discussion

This study allowed us to better understand and characterize Sinai Chicago's approach to introducing a novel vaccine to a community with high rates of COVID-19 infection and medical mistrust, educating members of the community, facilitating vaccine access, and finally to describe the characteristics of vaccinated patients over a 10-month period.

Our study is among the first to document vaccine promotion strategies used by an urban hospital that serves historically under-resourced communities and offers several lessons learned. The hospital used a multi-prong strategy guided by cultural and community context, partner collaboration, and community engagement. Sinai Chicago's Marketing team along with SCI collaborated with partners with lived experience to develop content based on the needs and beliefs of the community. Pop-up vaccination sites and mobile clinics were established based on the priority communities defined by Protect Chicago Plus.

As a result of the community-driven strategies and other external factors, over 11,000 patients received at least one dose of Pfizer or Moderna between January and October 2021 at a Sinai Chicago facility. We found a statistically significant difference in the odds of full vaccination by race/ethnicity. Compared to Non-Hispanic White patients, Non-Hispanic Black patients had a statistically significant lower odds of being fully vaccinated, and Hispanic patients has a statistically significant higher odds of being fully vaccinated. This may be explained by the lower number of Non-Hispanic White patients in our sample. However, national data from October to November 2021 found that Non-Hispanic Black, Hispanic, and Asian individuals are getting vaccinated at a slightly higher rate compared to Non-Hispanic White people (17). Similar to national (18) and city (19) data, we found that older individuals have a statistically significant higher odds of being fully vaccinated compared to those 18–24 years of age. This is important information to document to drive the ongoing prioritization of educating and improving access to individuals under the age of 24, particularly as the vaccine becomes available for younger age groups.

Our findings bring to light several lessons learned that can be applied for a more successful approach to future implementation of new health interventions. First, and most broadly, implementation should center the views, needs, and perspectives of the target population. Sinai was fortunate to be able to draw on many existing relationships given its position in the community and the long-standing work of SCI. The Marketing strategy was able to give voice to and therefore better understand the motivations of the community residents. For those doing similar work without a strong standing in the community, it is imperative to focus on collaboration with respected community partners in order to achieve a community-informed perspective. Second, implementation should be data-driven from the onset and throughout. This is one area in which Sinai could have improved its roll-out strategy. While city- and even community-level data were used to inform the roll-out, patient data from the EMR could have been examined along the way to highlight areas of additional need—either for specific racial/ethnic groups or by gender, age, or insurance status.

### Limitations

This study has a few limitations. First, we are not able to make causal claims between Sinai Chicago's COVID-19 vaccination education and access plan and the rates of vaccination among patients. Second, our informant interviews focused on operations and processes rather than outcomes of the vaccination plan. We did not speak to patients and therefore do not have the perspective from consumers of the marketing strategies used. Third, our quantitative vaccination data does not speak to community-level vaccine uptake because the denominator is vaccinated patients, not the vaccine-eligible community population. Finally, our data are specific to an under-resourced urban patient population and are not more widely generalizable. However, our study contributes important information on this population from a hospital patient perspective.

## Conclusion

Our study documents the innovative and multi-faceted roll-out of COVID-19 vaccine among an under-resourced urban area in Chicago and suggests important lessons learned for future implementation of health interventions, including vaccine and booster shots.

## Data Availability Statement

The raw data supporting the conclusions of this article will be made available by the authors, without undue reservation.

## Ethics Statement

The studies involving human participants were reviewed and approved by the Mount Sinai Hospital Institutional Review Board (protocol #21–47). Written informed consent for participation was not required for this study in accordance with the national legislation and the institutional requirements.

## Author Contributions

LD led the literature review and write-up of the Introduction, as well as the collection, analysis and write-up of the qualitative data. AB conducted the quantitative analysis for this study and led the writing of the abstract and quantitative Results section. BRH conceived of this study, helped to conceptualize the research questions, and led the writing of the Methods section. JJ conceived of this study, helped to conceptualize the research questions, and led the writing of the Discussion section. KJ contributed to the literature review and write-up of the Introduction section. AKJ guided and oversaw the analytical methods of this study and led the writing of the Introduction Section. She contributed to the writing of the Introduction, Discussion, and Conclusion sections. All authors contributed to the article and approved the submitted version.

## Conflict of Interest

The authors declare that the research was conducted in the absence of any commercial or financial relationships that could be construed as a potential conflict of interest.

## Publisher's Note

All claims expressed in this article are solely those of the authors and do not necessarily represent those of their affiliated organizations, or those of the publisher, the editors and the reviewers. Any product that may be evaluated in this article, or claim that may be made by its manufacturer, is not guaranteed or endorsed by the publisher.
